# Escherichia coli Bacteremia-induced Purpura Fulminans: A Case Report

**DOI:** 10.7759/cureus.3638

**Published:** 2018-11-26

**Authors:** Mohamed Ahmed, Michael Samotowka, Saba Habis, Ahmed Mahmoud, Rasha Saeed

**Affiliations:** 1 Surgery, Riverside Community Hospital / Envision Healthcare, Riverside, USA; 2 Surgery, Jackson Memorial Hospital, Jacksonville, USA; 3 Internal Medicine, Riverside Community Hospital / Hospital Corporation of America, Riverside, USA; 4 Surgery, Riverside Community Hospital / University of California, Riverside, USA

**Keywords:** purpura fulminans, escherichia coli, disseminated intravascular coagulation

## Abstract

Purpura fulminans (PF) is a dermatologic manifestation of an underlying life-threatening condition associated with disseminated intravascular coagulation and skin necrosis. The known categories include protein C deficiency or abnormalities of other coagulation systems (inherited or acquired), acute infectious PF and idiopathic. We describe a case of PF induced by *Escherichia coli-*associated bacteremia.

## Introduction

Purpura fulminans (PF) was first described by Guelliot in 1884 [1]. It is a life-threatening condition associated with disseminated intravascular coagulation (DIC) and skin necrosis that progresses rapidly to multi-system organ failure secondary to thrombotic occlusion of small- and medium-sized blood vessels [2]. Herein, we describe a case of intestinal *Escherichia coli* -associated bacteremia as a part of PF syndrome. 

## Case presentation

**Figure 1:**
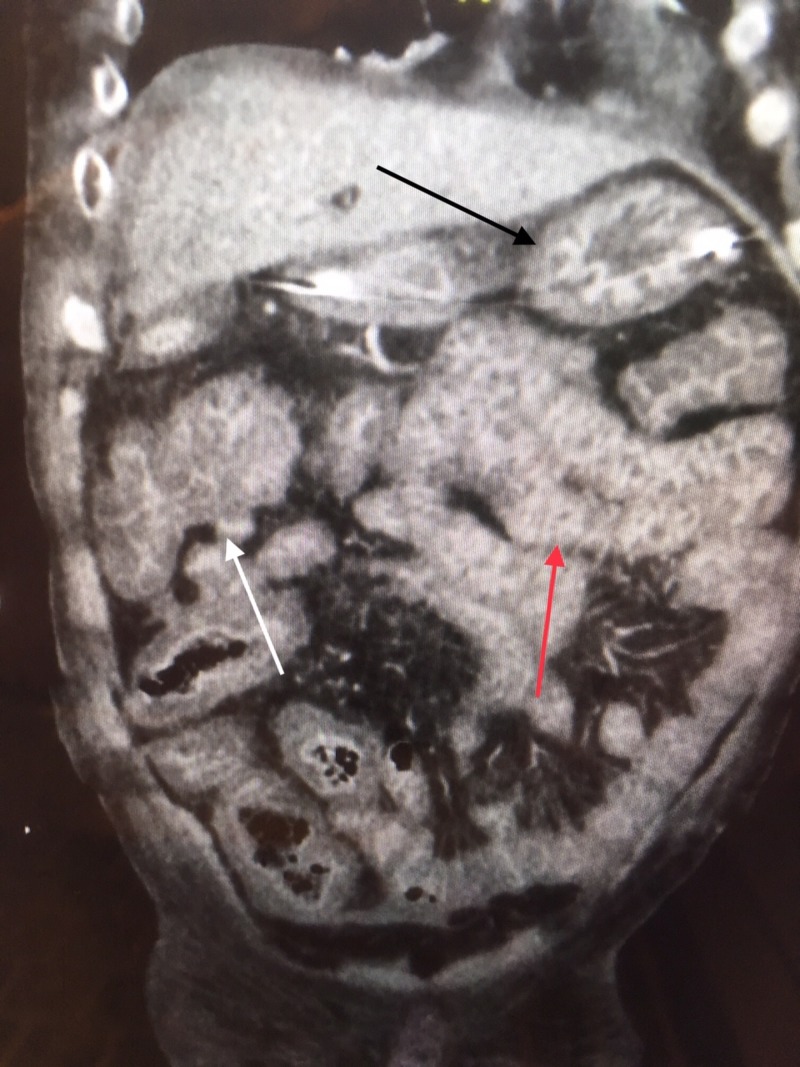
CT abdomen and pelvis Black arrow: inflamed stomach; white arrow: inflamed colon; red arrow: inflamed small bowel CT: computed tomography

A 62-year-old Hispanic male was admitted to the intensive care unit (ICU) with signs of septic shock. After an aggressive fluid resuscitation and administration of intravenous antibiotics including vancomycin and Zosyn, a computed tomography (CT) scan of the abdomen and pelvis was obtained revealing coloenteritis (Figure 1).

**Figure 2:**
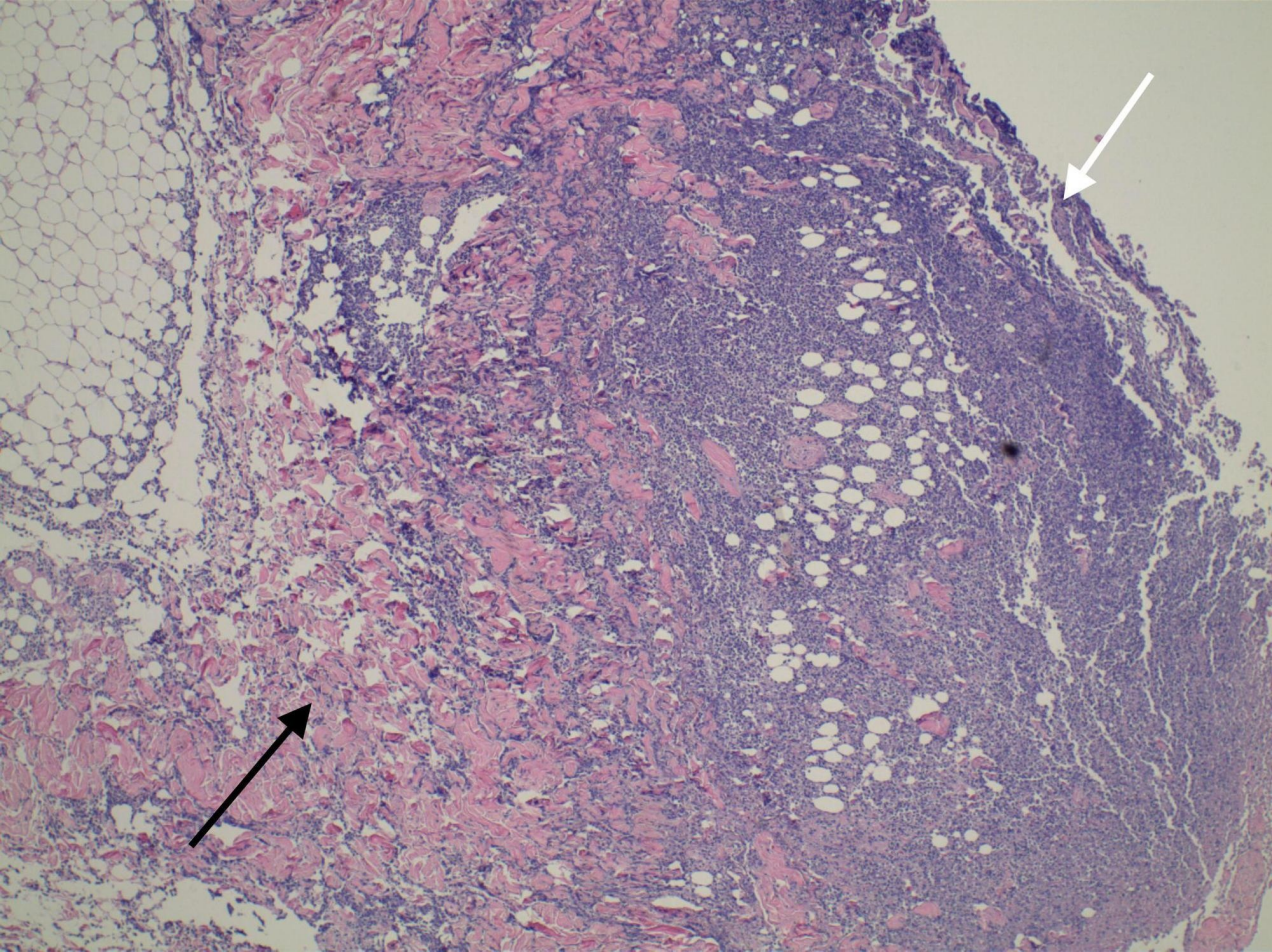
Skin histopathology White arrow: necrotic skin; black arrow: inflammatory changes

His course was complicated by DIC requiring transfusion of blood products, along with ventilatory support for hypoxic respiratory failure and beta-blocker medications for controlling the atrial fibrillation rate. Blood cultures grew *E.coli,* and antibiotic coverage was changed to meropenem and ceftriaxone. The patient developed bilateral flank dark-red discoloration and bullous lesions with copious weeping. Skin lesions progressed rapidly to full-thickness necrosis in spite of local wound care with topical silver sulfadiazine (Figure 2).

**Figure 3:**
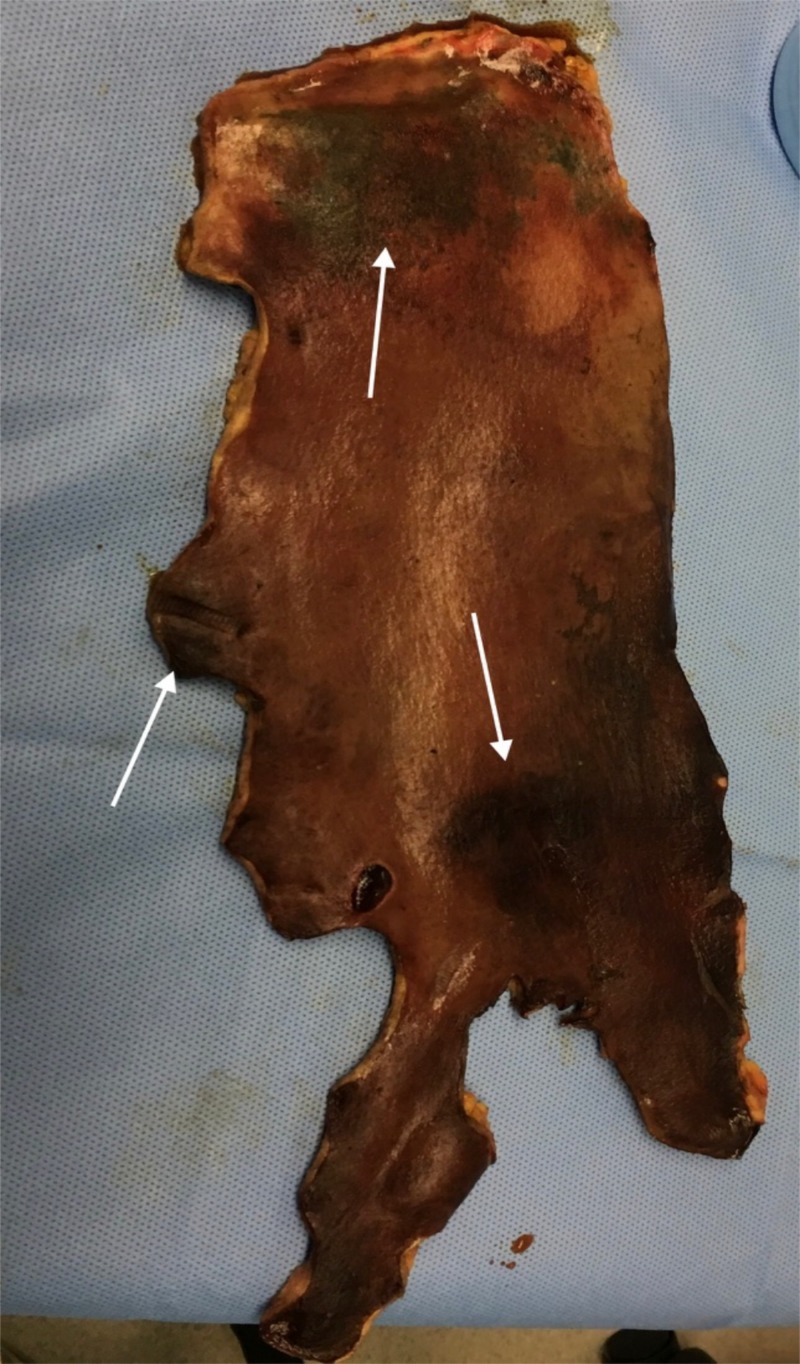
Excised necrotic skin White arrow: necrotic skin

The differential diagnoses (Coumadin-induced necrosis, thrombotic thrombocytopenic purpura, meningococcemia, toxic shock syndrome, calciphylaxis, necrotizing fasciitis and meningococcemia) were all ruled out. Necrotic skin and subcutaneous tissues required multiple surgical excisions, debridement and the use of a wound vacuum (Figure 3).

**Figure 4:**
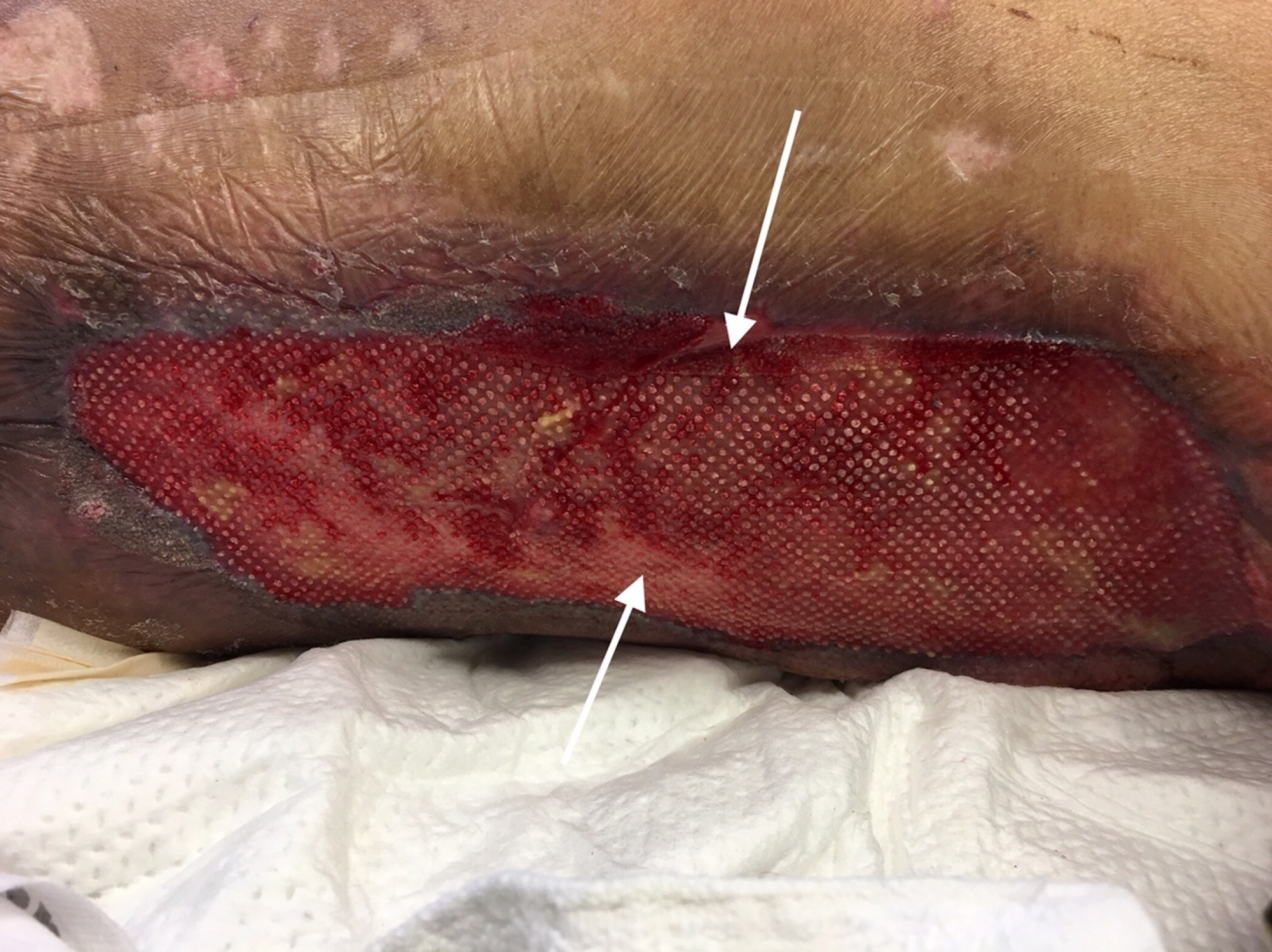
Skin grafting White arrow: skin graft

After a prolonged hospital stay with multidisciplinary care, local wound care and skin grafting, the patient did well and was discharged to an acute rehabilitation center (Figure 4).

## Discussion

PF is a cutaneous manifestation of an underlying sudden and severe DIC. Retiform purpura and skin necrosis develop as a result of the coagulopathies typical of DIC. The pathogenesis probably involves acute transient decreases in the levels of protein C, protein S, or antithrombin III (ATIII). It is categorized into protein C deficiency or abnormalities of other coagulation systems (inherited or acquired) abnormalities, acute infectious PF and idiopathic. Inherited causes are secondary to homozygous protein C or S deficiency although heterozygous has also been described. Antecedent infections commonly include *Neisseria meningitidis*, group A *Streptococcus, Staphylococcus, Pneumococcus,* *Vibrio, Meningococcus* and *Varicella*; however; *E. coli-*associated bacteremia has been reported [3-4]. Although *E.coli *is typically not an antecedent infectious agent, both intestinal [5] and extra-intestinal infections caused by *E.coli *have been reported [6-7]. Early recognition and treatment of PF are essential to reduce mortality, and management is tailored to the individual patient. It involves supportive therapy and replacement of blood products and clotting factors as appropriate, and in cases of septicemia, aggressive resuscitation and antibiotics are important [8-9]. Heparin may reverse skin necrosis, protein C may contribute to improved survival, antithrombin III may reverse DIC [10], recombinant tissue plasminogen activator (rtPA) can improve peripheral perfusion [11], topical nitroglycerin may improve skin blood flow and pain, plasmapheresis removes circulating endotoxin and assists in controlling fluid balance, epidural sympathetic block with local anesthetic can improve skin perfusion [12], necrotic skin and tissues can be managed by excisional debridement and local wound care and in cases of extensive loss, skin grafting is performed [13].

## Conclusions

PF is a morbid and potentially fatal condition that can be a cutaneous manifestation of *E. Coli* bacteremia. Early recognition and accurate identification of the underlying cause minimize morbidity and mortality. Management is tailored to the individual patient and in our case, aggressive early resuscitation, intravenous antibiotics, correction of coagulopathy, necrotic skin excision and, later, grafting were required.
